# Pneumococcal Antibody Concentrations and Carriage of Pneumococci more than 3 Years after Infant Immunization with a Pneumococcal Conjugate Vaccine

**DOI:** 10.1371/journal.pone.0031050

**Published:** 2012-02-20

**Authors:** Adebayo K. Akinsola, Martin O. C. Ota, Godwin C. Enwere, Brown J. Okoko, Syed M. A. Zaman, Mark Saaka, Ekpedeme D. Nsekpong, Aderonke A. Odutola, Brian M. Greenwood, Felicity T. Cutts, Richard A. Adegbola

**Affiliations:** 1 Medical Research Council (MRC), The Gambia Unit, Banjul, The Gambia; 2 London School of Hygiene and Tropical Medicine, London, United Kingdom; University of Witwatersrand, South Africa

## Abstract

**Background:**

A 9-valent pneumococcal conjugate vaccine (PCV-9), given in a 3-dose schedule, protected Gambian children against pneumococcal disease and reduced nasopharyngeal carriage of pneumococci of vaccine serotypes. We have studied the effect of a booster or delayed primary dose of 7-valent conjugate vaccine (PCV-7) on antibody and nasopharyngeal carriage of pneumococci 3–4 years after primary vaccination.

**Methodology/Principal Findings:**

We recruited a subsample of children who had received 3 doses of either PCV-9 or placebo (controls) into this follow-up study. Pre- and post- PCV-7 pneumococcal antibody concentrations to the 9 serotypes in PCV-9 and nasopharyngeal carriage of pneumococci were determined before and at intervals up to 18 months post-PCV-7. We enrolled 282 children at a median age of 45 months (range, 38–52 months); 138 had received 3 doses of PCV-9 in infancy and 144 were controls. Before receiving PCV-7, a high proportion of children had antibody concentrations >0.35 µg/mL to most of the serotypes in PCV-9 (average of 75% in the PCV-9 and 66% in the control group respectively). The geometric mean antibody concentrations in the vaccinated group were significantly higher compared to controls for serotypes 6B, 14, and 23F. Antibody concentrations were significantly increased to serotypes in the PCV-7 vaccine both 6–8 weeks and 16–18 months after PCV-7. Antibodies to serotypes 6B, 9V and 23F were higher in the PCV-9 group than in the control group 6–8 weeks after PCV-7, but only the 6B difference was sustained at 16–18 months. There was no significant difference in nasopharyngeal carriage between the two groups.

**Conclusions/Significance:**

Pneumococcal antibody concentrations in Gambian children were high 34–48 months after a 3-dose primary infant vaccination series of PCV-9 for serotypes other than serotypes 1 and 18C, and were significantly higher than in control children for 3 of the 9 serotypes. Antibody concentrations increased after PCV-7 and remained raised for at least 18 months.

## Introduction

*Streptococcus pneumoniae* (the pneumococcus) is estimated to cause nearly one million childhood deaths each year [1]. Most of these deaths occur in developing countries where the pneumococcus is the most frequent cause of childhood pneumonia and where mortality from pneumococcal meningitis is high (around 50%) with many survivors left with severe neurologic disabilities [2], [3]. There is a high burden of pneumococcal disease in The Gambia [4], [5] where the pneumococcus is the most prevalent bacterial pathogen isolated from children with pneumonia and is responsible for about 50% of cases of pyogenic meningitis [3], [4], [6]. About 40% of the serogroups responsible for invasive disease in young children in The Gambia are covered by the 7-valent pneumococcal conjugate vaccine (Prevenar^R^, Pfizer) and about 80% by the 9-valent pneumococcal conjugate vaccine used in trials in The Gambia and South Africa [4], [5], [7], [8].

Pneumococcal conjugate vaccines prevent invasive pneumococcal diseases (IPD) both directly and indirectly by reducing transmission [9], [10]. The 9-valent pneumococcal conjugate vaccine (PCV-9) given in a 3-dose schedule beginning at 6 weeks of age, with a minimum of 4-week intervals between doses, induced protective levels of anti-pneumococcal antibodies [11] and provided protection against IPD, pneumonia and all-cause mortality in Gambian children up to the end of follow-up at age 30 months [12]. Antibody concentrations with conjugate vaccines decline after primary vaccination. The rate of decline and the persistence of immunologic memory are important parameters in determining the potential need and time for booster vaccination [13]. Gambian children who received primary vaccination with 2 or 3 doses of a 5-valent PCV in infancy showed immunologic memory at 24 months of age [14], but there are few data on declines in antibody concentration or on the persistence of immunologic memory beyond this period in children in developing countries.

The currently recommended regimen for PCV in the United States is to follow primary immunization at 2, 4 and 6 months of age with a booster dose in the second year of life [15]. The high prevalence of nasopharyngeal carriage in developing countries such as The Gambia could provide natural boosting such that the kinetics of the antibody response to PCV could differ from that seen in developed countries and make a booster dose unnecessary, with important cost savings for countries with limited resources. To inform international policy on whether there is a need for booster immunization in low-income countries, more information is needed on the longevity of the antibody response following primary immunization in settings where pneumococcal carriage and diseases are common. We have, therefore, investigated the persistence of pneumococcal antibodies more than 3 years after primary vaccination in early infancy in children who had previously participated in the Gambian Pneumococcal Vaccine Trial (PVT) [12].

## Methods

### Setting and recruitment of study participants

The subjects who participated in this study had previously taken part in a double blind, placebo-controlled, individually randomized trial of PCV-9 that took place in The Gambia between 2000 and 2004 [12]. This trial enrolled 17,437 children, who received three doses of either PCV-9 (vaccinated group) or placebo (control group). The primary immunization schedule adopted for this trial was vaccination at 6, 10 and 14 weeks of age but due to the rural setting, the median age at receipt of the first dose of vaccine or placebo was 11 weeks (inter quartile range [IQR] 8–16 weeks) and for the third dose it was 24 weeks (IQR 19–32 weeks) [12]. After the trial results were reviewed, Wyeth vaccines kindly donated 7-valent PCV vaccine (PCV-7) for all children in the study area in the age cohort that would have been eligible to participate in the trial. A week- long vaccination campaign with PCV-7 was organized by the Gambian Ministry of Health in Upper and Central River Regions in June 2005 for all children aged 2–4 years, and approximately 27,000 children (an estimated 87% of the eligible total) were vaccinated with one dose, which served as booster dose for children who had previously received PCV-9 and as delayed primary immunization for the control group. A subset of participants from the vaccinated group was selected for the current evaluation of antibody persistence and response to booster vaccination with PCV-7, and the impact of delayed primary immunization with PCV-7 was studied in a subset of children in the previous control group. Nasopharyngeal carriage of pneumococci was studied in both groups.

A list was generated of subjects aged 3–4 years who had received three doses of PCV-9 or placebo during the PCV-9 trial and who lived near to one of a selected number of health centres to allow for ease of follow up. Children for participation in the follow-up study were recruited sequentially from this list until the required sample size had been reached. The study participants were not age or sex matched. Separate consent for participation of children selected for this extended study was obtained from parents/guardians of the participants before enrollment. The field, clinical and laboratory investigators were blind to the group of the study participants. The study was approved by the Joint Gambian Government/MRC Ethics Committee.

### Enrolment, follow up and sampling

**Figure 1:**
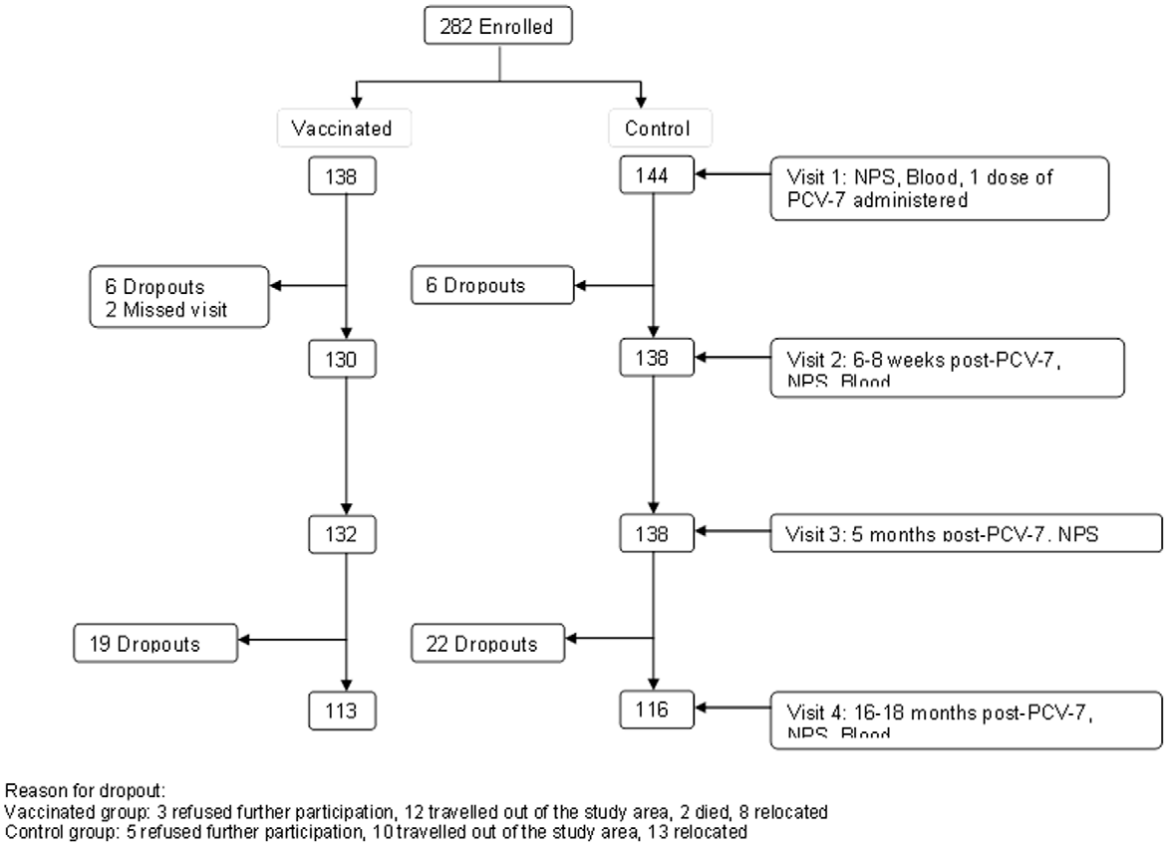
A consort diagram indicating number of study participants per study group and visit.

At enrolment into the follow-up study in late May 2005, a 3 ml venous blood sample and a nasopharyngeal swab were collected. The calcium alginate fibre tip of the applicator swab was cut off, placed in a container of skim milk-tryptone-glucose-glycerin (STGG) transport medium, and held frozen at −70°C until analyzed. Serum was separated and kept frozen at −20°C until analysis. A full dose of PCV-7 (Pfizer) was given during the campaign from 8–15 June 2005. Study participants were seen at 6–8 weeks, 5 months and 16–18 months following booster/delayed primary PCV-7 vaccination when further samples were taken for determination of antibody concentrations (at 6–8 weeks and 16–18 months) and carriage of pneumococci (all visits) (Figure 1).

### Laboratory methods

Serology: Concentrations of serotype-specific anti-pneumococcal polysaccharide IgG antibodies were measured by enzyme linked immunoassay (ELISA). Test samples and controls were tested for type-specific IgG antibodies to the nine-polysaccharides in PCV-9 (1, 4, 5, 6B, 9V, 14, 18C, 19F, and 23F) according to an adapted WHO protocol as described previously [11].

Microbiology: Vials containing a tip of a nasopharyngeal swab (NPS) in transport medium were thawed to room temperature and 100 µl of the sample were diluted ten-fold with sterile Tryptone Soya Broth (TSB). 100 µl of broth were plated onto a selective gentamicin blood agar plate, which was incubated for 18–24 hours at 35°C in an atmosphere containing 5% carbon dioxide. Aliquots of isolates in 15% glycerol broth were kept frozen at −70°C for future analysis. Identification of pneumococci was based on cultural morphology, susceptibility to ethylhydrocupreine hydrochloride (optochin) and sodium deoxycholate (bile solubility). Serotyping was performed with capsular and factor-typing sera (Statens Serum Institut, Copenhagen, Denmark), using a latex agglutination assay as described previously [16]. Isolates with equivocal results were confirmed by the Quellung reaction. *S. pneumoniae* (ATCC 49619) was used as a quality control strain. All laboratory assays were done blinded to the child's study group.

### Statistical analyses

To give a study with 90% power (alpha 0.05) to detect a 30% reduction in carriage of pneumococci of vaccine serotype in infants who had received PCV-9 in infancy at the time of booster immunization, assuming an NP carriage rate of pneumococci of vaccine serotype of 75% based on earlier small studies in non-vaccinated children [14], [17] a sample size of 110 children per group was needed. This was increased to allow for losses to follow-up. Differences in antibody concentrations between the groups were determined by comparing the geometric means, as well as the proportions of children with an antibody concentration of ≥0.35 µg/mL, the concentration considered to be protective against IPD [18]. Geometric means of the antibody concentrations in the two groups were compared by the two-sample t-test. For categorical variables, groups were compared using the Chi-Squared test, or for small numbers, Fisher's exact test. The Holm's method was used to adjust the P-values for multiple comparisons between groups [19].

## Results

We recruited 284 participants and obtained adequate blood samples from 282, including 138 previously vaccinated with PCV-9 and 144 controls. The mean age at first visit for the follow-up study was 45 months. Age, gender and area of domicile were similar between the two study groups (data not shown). At the final visit 16–18 months after PCV-7, 113 (82%) and 116 (81%) children from vaccinated and control groups respectively were evaluated. There was no significant difference in the number of children who dropped out of the study between the groups, neither was there any difference in the reasons for failure to participate further in the study (Figure 1). The period that had elapsed between the last dose of PCV-9 or placebo and receipt of PCV-7 ranged from 34 to 48 months.

### Anti-pneumococcal antibody concentrations before PCV-7 vaccination

**Figure 2:**
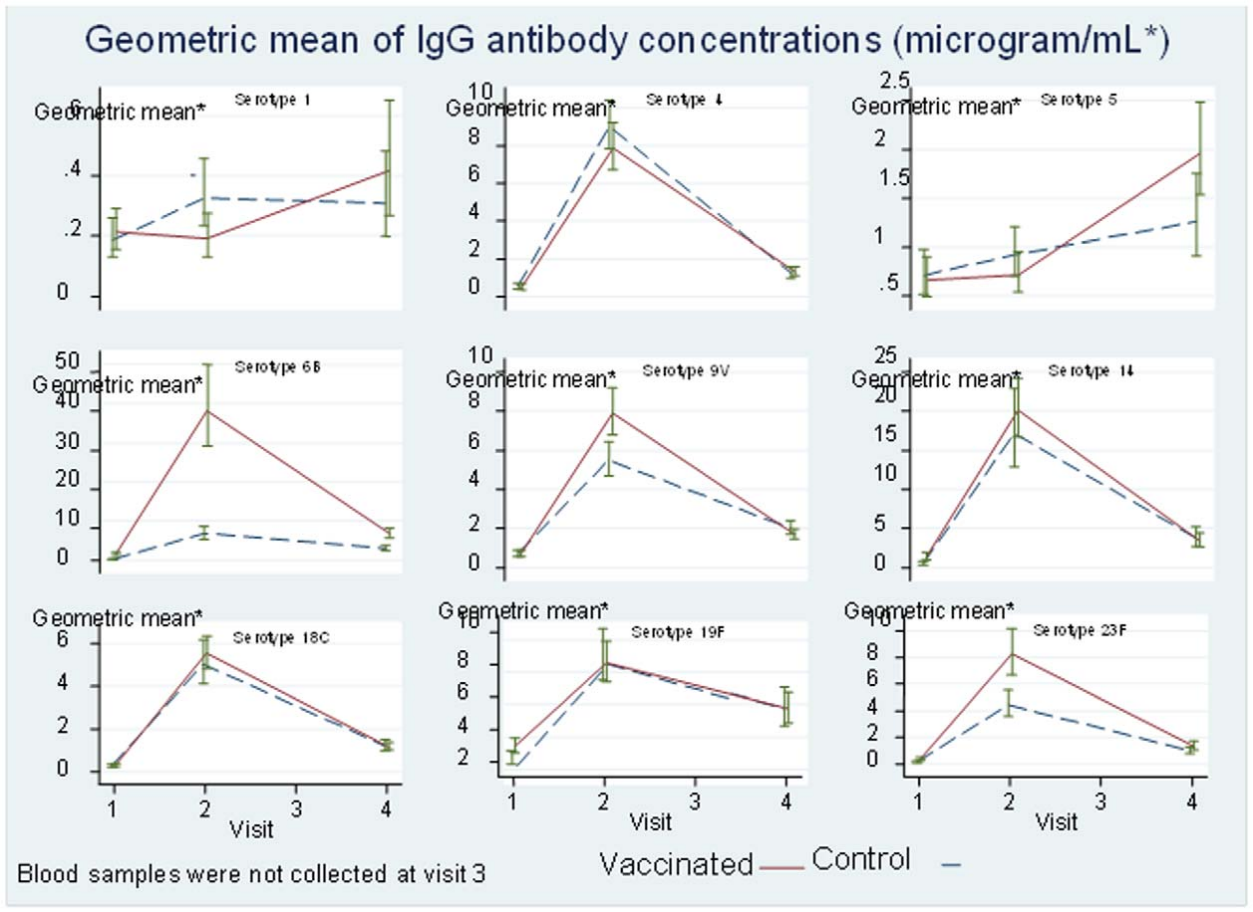
Geometric mean of IgG antibody concentrations (µg/mL) before and after vaccination with PCV-7 in the two groups (visit 1 = pre-vaccination).

The baseline antibody concentrations before booster/delayed primary vaccination with PCV-7 were high for most of the PCV-9 serotypes, although lower than those reported 4-weeks after administration of the third dose of PCV-9 in the original trial [11] (Table 1). The average (range) of the proportions of children who had protective antibody concentrations (≥0.35 µg/mL) for each serotype at baseline was 75.0% (47.4–99.3%) and 65.9% (36.8–96.5%) for the vaccinated and control children respectively (Table 2). There was considerable variation in the mean antibody concentration seen for individual polysaccharides (Table 1, Fig. 2) with the lowest concentration (and lowest proportion having protective antibody concentrations) being found for antibodies to serotype 1 in both groups. Serotype 6B, 14 and 23 antibody concentrations were significantly higher in previously vaccinated than in control children (Table 1).

**Table 1:** Geometric means (95% CI) of IgG antibody concentrations (µg/mL) before and after boosting with PCV-7 in the group that previously had placebo (Control) or PCV-9 (Vaccinated).

	Before vaccination with PCV-7		6–8 weeks post-PCV-7 (visit 2)		16–18 months post-PCV-7 (visit 4)	
Serotype	Control	Vaccinated	Control	Vaccinated	Control	Vaccinated
In PCV-9 only						
1	0.095 (0.057–0.157)	0.130 (0.082–0.205)	0.206 (0.126–0.335)	0.094 (0.054–0.164)	0.183 (0.099–0.338)	0.294 (0.165–0.523)
5	0.510 (0.328–0.793)	0.502 (0.331–0.762)	0.759 (0.523–1.101)	0.573 (0.384–0.855)	1.045 (0.670–1.632)	1.862 (1.401–2.474)
In PCV-7 and PCV-9						
4	0.398 (0.263–0.603)	0.352 (0.227–0.547)	9.009 (7.808–10.394)	7.864 (6.709–9.218)	1.125 (0.810–1.561)	1.301 (1.056–1.602)
6B	0.092 (0.056–0.150)	1.485* (1.09–2.02)	6.688 (5.112–8.750)	38.181* (29.136–50.034)	2.881 (2.199–3.773)	6.814 (5.662–8.201)
9V	0.626 (0.454–0.857)	0.647 (0.479–0.875)	5.514 (4.700–6.468)	7.912* (6.803–9.202)	2.006 (1.701–2.366)	1.733 (1.514–1.984)
14	0.320 (0.188–0.547)	1.127* (0.720–1.766)	16.553 (12.039–22.760)	20.097 (16.680–24.213)	3.584 (2.489–5.161)	3.348 (2.461–4.556)
18C	0.173 (0.112–0.267)	0.213 (0.143–0.318)	4.935 (3.958–6.154)	5.520 (4.803–6.345)	1.136 (0.869–1.486)	1.172 (1.009–1.362)
19F	2.168 (1.76–2.68)	2.968 (2.55–3.46)	8.347 (6.793–10.257)	8.074 (6.924–9.415)	5.195 (4.060–6.647)	5.256 (4.424–6.245)
23F	0.094 (0.061–0.146)	0.305* (0.209–0.445)	4.376 (3.452–5.549)	8.243* (6.669–10.188)	0.911 (0.650–1.277)	1.297 (0.981–1.715)

*The GMs of antibodies to these serotypes were significantly higher in the vaccinated group than the controls.

**Table 2:** Proportions of children aged 3–4 years with antibody concentration ≥0.35 µg/mL before and after vaccination with PCV-7.

	Baseline (visit 1)			6–8 weeks post-PCV-7 (visit 2)			16–18 months post-PCV-7 (visit 4)		
Serotype	Vaccinated	Control	p-value	Vaccinated	Control	p-value	Vaccinated	Control	p-value
In PCV-9 only	n(% pos)	n(% pos))		n (% pos)	n (% pos)		n(% pos)	n (%pos)	
1	135 (47)	141 (52)	0.473	124(54)	130(63)	0.143	96(70)	104(61)	0.127
5	137 (82)	144 (82)	0.906	122(86)	130(89)	0.445	97(98)	102(91)	0.851†
In PCV-7 and PCV-9									
4	138 (67)	144 (72)	0.436	128 (100)	135(100)	1.000	102(97)	110(92)	0.099
6B	134 (91)	142 (47)	<0.0001	94(99)	125(97)	0.295	104(100)	112(96)	0.696
9V	137 (84)	143 (80)	0.439	128(100)	136(100)	1.000	102(98)	109(97)	0.706
14	138 (88)	144 (72)	0.023*	129(100)	130(98)	0.157	105(98)	108(97)	0.674
18C	138 (55)	144 (56)	0.935	129(100)	137(99)	0.168	96(95)	111(96)	0.814
19F	138 (99)	144 (96)	0.215	129(100)	137(99)	0.331	106(100)	111(99)	0.327
23F	138 (60)	144 (37)	0.003**	124(99)	134(98)	0.352	98(94)	100(85)	0.946‡

*P value = 0.0009 before Holm's correction for multiple significance tests.

**P value = 0.0001 before Holm's correction for multiple significance tests.

† P value = 0.037 before Holm's correction for multiple significance tests.

‡ P value = 0.043 before Holm's correction for multiple significance tests.

### Antibody response to booster/delayed primary vaccination with PCV-7

The proportions of children who achieved antibody concentrations ≥0.35 µg/mL were high 6–8 weeks after booster/delayed primary vaccination with PCV-7, ranging from 97–100% for individual PCV-7 serotypes (Table 2). There were no significant differences in the proportions with “protective” antibody concentrations to polysaccharides in PCV-7 (4, 6B, 9V, 14, 18C, 19F and 23F) between groups at 6–8 weeks or at 16–18 months post-vaccination, after accounting for multiple significance tests. However, the geometric mean antibody concentration in the PCV-9 group was higher than in the control group for serotypes 6B, 9V and 23F at 6–8 weeks but only for serotype 6B at 16–18 months after boosting (Fig. 2 and Table 1).

Fig. 2 shows the kinetics of the antibody response following booster/delayed primary vaccination with PCV-7. For the PCV-7 serotypes, antibody concentrations increased markedly at 6–8 weeks after vaccination with PCV-7 (Visit 2) but then fell sharply in both groups, though remaining higher than pre-PCV-7 levels at 16–18 months post-vaccination. For serotypes 1 and 5 which are in PCV-9 but not PCV-7, GM concentrations at 6–8 weeks were no higher than pre-boost in the PCV-9 group, but had increased at 16–18 months post-boost; concentrations in the control group increased over time.

### Nasopharyngeal carriage

The proportions of study children who carried pneumococci of each of the PCV-7 serotypes, and serotypes 1 and 5, present in PCV-9, are shown at different time-points post-vaccination for the two study groups in Table 3. The frequency of nasopharyngeal carriage of each individual serotype was too low to allow meaningful individual comparisons, and no child carried types 1 or 5 at any time point post-vaccination. Thus, further comparisons were done for carriage of any PCV-9 serotype, PCV-9-related types, and non-PCV-9 serotypes between the groups. No significant differences were found in the proportions of nasopharyngeal carriers in these categories between the two groups at any time point. The tendency for children in the vaccinated group to carry more non-vaccine serotypes (39%) than children in the control group (28%) remained at 6–8 weeks post-vaccination but not at 5 months post-PCV-7 or later.

**Table 3:** Nasopharyngeal carriage of *Streptococcus pneumoniae* before and after vaccination with a single dose of PCV-7.

Serotype	Baseline (visit 1)			6–8 weeks post-PCV-7 (visit 2)			5 months post-PCV-7 (visit 3)			16–18 months post-PCV-7 (visit 4)		
	Vaccinated (N = 137)* n (%)**	Control (N = 143) n (%)	P value	Vaccinated (N = 129) n (%)	Control (N = 138) n (%)	P value	Vaccinated (N = 131) n (%)	Control (N = 138) n (%)	P value	Vaccinated (N = 106) n (%)	Control (N = 99) n (%)	P value
1	0	1 (0.7)	1.00	0	0	NA	0	0	NA	0	0	NA
4	1 (0.7)	2 (1.4)	1.00	0	0	NA	1 (0.8)	0	0.487	0	0	NA
5	0	0	NA	0	0	NA	0	0	NA	0	0	NA
6B	2 (1.5)	7 (5)	0.174	1(0.8)	4(3)	0.372	1 (0.8)	6 (4)	0.121	0	1(1)	0.483
9V	1 (0.7)	0	0.489	1(0.8)	0	0.483	0	0	NA	0	1(1)	0.483
14	3 (2)	4 (3)	1.00	1(0.8)	2(1.5)	1.000	2 (1.5)	1 (0.7)	0.614	0	0	NA
18C	0	0	NA	0	1(0.7)	1.000	0.000	1 (0.7)	1.000	2(2)	0	0.498
19F	8 (6)	3 (2)	0.131	8(6)	4(3)	0.243	5 (4)	3 (2)	0.491	1(1)	0	1.000
23F	3 (2)	7 (5)	0.336	3(2)	4(3)	1.000	4 (3)	3 (2)	0.717	3(3)	0	0.247
All PCV9 types	18 (13)	24 (17)	0.393	14(11)	15(11)	1.000	13 (10)	13 (9)	0.889	6(6)	2(2)	0.282
All PCV9-related types	23 (17)	28 (20)	0.545	13 (10)	21(15)	0.208	16 (12)	14 (10)	0.57	9(8)	12(12)	0.392
All non-PCV9 types	53 (39)	40 (28)	1†	50(39)	37(27)	1‡	48 (37)	52 (38)	0.86	30(28)	26(26)	0.756

NA = Not applicable.

*Number of children.

**Number of serotypes (some children had multiple serotypes on a single visit: they have been included in all those serotype groups).

† P value = 0.057 before Holm's correction for multiple significance tests.

‡ P value = 0.037 before Holm's correction for multiple significance tests.

## Discussion

A substantial proportion of the children in the Gambian pneumococcal vaccine trial had antibody concentrations considered to be protective against invasive pneumococcal diseases for most of the serotypes investigated at the age of 3–5 years, regardless of whether or not they had received PCV-9, although proportions were a little higher overall for children who had previously been vaccinated with PCV-9 and substantially higher for some serotypes. The relatively high concentrations of anti-pneumococcal antibodies observed in our study population are similar to those demonstrated previously in PCV vaccinated Gambian children [11], [20], [21]. The significant differences in antibody concentrations prior to vaccination with PCV-7 for serotypes 6B, 14, and 23F between the groups indicate longevity of some vaccine induced antibodies. This may be a result of boosting by natural colonization and occult pneumococcal infection [22]. However, it is of concern that antibody concentrations to serotype 1 polysaccharide were the lowest among those measured, as pneumococci belonging to this serotype are a frequent cause of IPD in The Gambia and in other developing countries. The high antibody concentrations in the control group for serotypes 4, 9V, 14 and 19F probably reflect past carriage with these organisms. High antibody concentrations to serotype 5, which was not commonly found in carriage studies of infants in the PVT [23] nor in this follow-up study, may reflect past short-duration carriage which is less likely to be detected in cross-sectional studies, and/or past occult or symptomatic invasive infection. Previous studies of infants and toddlers in The Gambia have found carriage of serotypes 19F, 6B, 23F and 9V to be the most common among the vaccine-serotypes [23]. Similar vaccine serotypes were found to be the most common in another carriage study that cuts across all age groups including adults prior to national routine PCV-7 vaccination in the Western Region of the Gambia [24]. A high proportion of children with antibody concentrations of ≥0.2 µg/mL 5 years after primary vaccination with PCV-9 was described for serotypes 4, 6B, 9V, 18C, and 23F among HIV uninfected South African children [25]; in our study proportions were lower for serotypes 1, 4, 18C and 23F than for other PCV-9-serotypes. These data support the existence of differential host and/or environmental factors influencing the responses to the various serotypes contained within pneumococcal conjugate vaccines.

A strong response to PCV-7 at the age of 3–5 years was seen in children in both groups. The proportion of children with antibody concentrations considered to be protective against invasive pneumococcal disease more than 3 years after primary immunization ranged from 55 to 99% in the PCV-9 group and 47 to 96% in controls, but the booster/delayed primary vaccination increased this proportion to >90% for all PCV-7 serotypes in both groups for up to 18 months, except for serotype 23F in controls which had fallen to 85% at 18 months post-PCV-7. These increases in the antibody concentration following booster/delayed primary PCV-7 contribute to consideration of whether or not to include a booster dose in the PCV vaccination schedule in countries where carriage of pneumococci is common. Such a review of vaccination schedules would require a study with more frequent assessment of the kinetics of antibody concentrations to fully assess a potential anamnestic response among previously vaccinated children. We have shown recently that in resource-poor settings, administration of a booster dose of pneumococcal polysaccharide vaccine (PPV-23) following primary immunization with one or two doses of PCV-7 diminished the differences in initial antibody responses and might lower the cost [26]. Further study of optimal pneumococcal vaccination schedules would be helpful [27], [28]. The effect of a single dose of PCV-7 on antibody concentrations in the control group also supports the WHO recommendation that when PCV is first introduced into routine childhood immunization programmes a single catch-up dose of PCV-7 may be given to previously unvaccinated children aged 12–24 months and to children aged 2–5 years who are at high risk [27].

No significant differences were found in the proportions of children carrying pneumococci between the children who had received booster/delayed primary vaccination and the control group at any time point. Following booster or delayed primary immunization with PCV-7 there was a small decline in the prevalence of carriage with pneumococci of vaccine serotype as might have been expected, which was not seen for non-vaccine serotypes. The initial sample size calculation for this study was based on the assumption that the overall carriage rate of pneumococci of vaccine serotype in children in the control group would be 75%. Because this figure was only 17% there was minimal power to detect significant differences between groups. The increase in prevalence of carriage of pneumococci of non-vaccine type among children who had received PCV-9 in infancy previously reported [23] persisted to the age of 3–4 years and up to 6 weeks after the booster vaccination, but this difference was minimal at and after 5 months post-PCV-7 vaccination.

Our study was not designed to assess correlation between antibody levels and carriage. Another report showed high levels of functional antibody after post-primary PPV-23 vaccination without impact on carriage, although there had appeared to be an effect of the number of doses of conjugate vaccine received on carriage at age 9 months [29]. An earlier study of primary conjugate pneumococcal vaccination had found that higher IgG concentrations led to a decreasing probability of having a new acquisition of pneumococcal carriage of the corresponding serotype, and achieved statistical significance for serotypes 14 and 19F [30]. Among adults, a pneumococcal anticapsular IgG concentration of 5 ug/mL has been shown to correlate with protection against carriage of serotype 14 [31].

The prevalence of carriage before PCV-7 vaccination was lower than has been reported previously in other parts of The Gambia where overall carriage across a community including adults was 72%. It was 97% among children <1 year old and 93% among babies of ages <1 month [24], [32]. The lower than expected carriage of pneumococci before PCV-7 vaccination was surprising and may be in part related to temporal trends in pneumococcal carriage but also to the increasing age of the study population.

In conclusion, there were significantly higher antibody concentrations to 3 of the 9 serotypes in vaccinated children compared to controls approximately 3 years after primary vaccination with PCV-9, and antibody levels in PCV-9 recipients and controls were increased by PCV-7. Carriage of vaccine serotypes was low in both groups and we could not assess adequately the effect of PCV-7 on this endpoint.
